# Undercarboxylated osteocalcin correlates with insulin secretion in Japanese individuals with diabetes

**DOI:** 10.1186/s13098-020-00579-3

**Published:** 2020-08-17

**Authors:** Shogo Funakoshi, Kumiko Yoshimura, Seiki Hirano, Satoko Ohmi, Eri Amano, Yoshiharu Fukuda, Yoshio Terada, Shimpei Fujimoto

**Affiliations:** 1 Department of Endocrinology, Metabolism, and Nephrology, Kochi Medical School, Kochi University, Kohasu, Oko-cho, Nankoku, Kochi 783-8505 Japan; 2 Fukuda Clinic, Kochi, Kochi 780-0023 Japan

**Keywords:** Insulin secretion, BMI, Undercarboxylated osteocalcin

## Abstract

**Background:**

Undercarboxylated osteocalcin (ucOC) is a secreted protein produced by osteoblasts that regulates insulin secretion and insulin sensitivity in rodents. However, the significance of these effects on glucose metabolism in human remains unknown. Moreover, the pathophysiological roles of ucOC on varying degrees of glucose intolerance, including diabetes need to be elucidated. In the present study, correlations between ucOC and indices of insulin secretion and sensitivity were analyzed in normal glucose tolerance (NGT), impaired glucose metabolism (IGM), and diabetes mellitus (DM) groups.

**Methods:**

Based on 75 g OGTT data in Japanese individuals without diabetic medication, or medications which may affect ucOC levels, individuals were classified as having normal glucose tolerance (NGT), impaired glucose metabolism (IGM), or diabetes (DM). In each group, 25 individuals were consecutively recruited [total 75 individuals, age: 65 ± 11 (mean ± SD); BMI: 24.9 ± 3.8 kg/m^2^]. QUICKI and Matsuda index (MI) were calculated as insulin sensitivity indices. Homeostasis model assessment (HOMA)-β and insulinogenic index (IGI) were calculated as insulin secretion indices. UcOC was measured using ECLIA. Normally-distributed log_e_-transformed (ln-) values were used for ucOC, HOMA-β, IGI, and MI.

**Results:**

The ucOC was not significantly different among the three groups. The results of multiple regression analysis showed that ln-ucOC did not significantly correlate with age, sex, BMI, waist circumference, fasting plasma glucose, plasma glucose 120 min after glucose loading, fasting plasma immunoreactive insulin, ln-HOMA-β, QUICKI, or ln-MI in any of the three groups. Interestingly, ln-ucOC correlated with ln-IGI (r = 0.422, *P* = 0.0354) and HbA1c (r = − 0.574, *P* = 0.0027) only in the DM group. There was no significant correlation between ln-IGI and age, sex, BMI, or HbA1c in the DM group. Further, the results of multiple regression analysis showed that ln-IGI could be independently predicted by BMI (β = 0.598, P = 0.0014) and ln-ucOC (β = 0.641, P = 0.0007) in the DM group (R^2^ = 0.488, *P* = 0.0006).

**Conclusion:**

In our study, ucOC positively correlated with insulin secretion independently of BMI in Japanese individuals with diabetes. These results suggest that ucOC plays more important roles in insulin secretion than in insulin sensitivity in individuals with diabetes.

## Background

In recent years, evidences from studies on mice have suggested that bone is a key organ that modulates glucose metabolism [1–3]. Osteocalcin (OCN), a known biomarker of bone turnover which is a unique protein synthesized by osteoblasts [2], plays important roles in glucose metabolism, since OCN-deleted mice exhibited hypoinsulinemia, hyperglycemia, glucose intolerance and insulin resistance [1].

In murine studies, these function of OCN are fulfilled by its undercarboxylated form, namely undercarboxylated osteocalcin (ucOC) [2]. G protein-coupled receptor C6A (GPRC6A), found in the Leydig cells of the testis, is also expressed in pancreatic β-cells [4]. In mouse β cells, ucOC promotes proliferation via GPRC6A and increases insulin secretion [2, 4]. UcOC also promotes β cell proliferation and insulin secretion in human pancreatic islets [5]. Osteocalcin exerts a favorable influence on insulin sensitivity in mice through a poorly understood mechanism [1, 2]. Recently, it is revealed that OCN signaling via GPRC6A favors glucose uptake and utilization by myofibers [6].

In cross-sectional studies, higher serum levels of OCN were negatively related to fasting plasma glucose (FPG) and glycated hemoglobin A1c (HbA1c) in individuals with mainly normal and impaired glucose tolerance [7–9]. Although basic studies have indicated that ucOC affects both insulin secretion and insulin sensitivity, the significance of these effects on glucose metabolism in human remains unknown. Moreover, the pathophysiological roles of ucOC on varying degrees of glucose intolerance, including diabetes need to be elucidated. In the present study, correlations between ucOC and indices of insulin secretion and sensitivity were analyzed in normal glucose tolerance (NGT), impaired glucose metabolism (IGM), and diabetes mellitus (DM) groups.

## Methods

### Participants

We analyzed data derived from a 75 g oral glucose tolerance test (75gOGTT) involving 75 Japanese individuals who did not take any diabetic medication [sex (M/F): 34/41; age: 65 ± 11 (mean ± SD); BMI: 24.9 ± 3.8 kg/m^2^], as described previously [10]. Participants did not take warfarin and any anti-osteoporosis medication including vitamin D, vitamin K, and bisphosphonate. The study protocol was approved by the Ethical Review Board of Kochi Medical School, Japan.

### Laboratory examinations and anthropometric data

Based on 75gOGTT data, individuals were either classified as NGT, IGM, or DM according to the 2006 World Health Organization (WHO) criteria [11] [NGT, fasting plasma glucose (FPG) < 110 mg/dL and 2-h plasma glucose (2-h PG) < 140 mg/dL; IGM, either impaired fasting glucose (IFG, FPG ≥ 110 and < 126 mg/dL) and/or impaired glucose tolerance (IGT, 2-h PG ≥ 140 and < 200 mg/dL); DM, FPG ≥ 126 mg/dL and/or 2-h PG ≥ 200 mg/dL].Fasting serum levels of ucOC were measured using a ucOC electro-chemiluminescence immunoassay (ECLIA) Kit (Picolumi ucOC, Eidia Co., Ltd., Tokyo). Although fasting plasma glucose, plasma glucose 120 min after glucose load, and HbA1c were significantly different among the 3 groups, age, sex, BMI were not significantly different as previously described [10].

### Indices of insulin secretion and insulin sensitivity

Indices of insulin secretion [homeostasis model assessment (HOMA)-β and insulinogenic index (IGI)] and insulin sensitivity [Quantitative Insulin Sensitivity Check Index (QUICKI) and the Matsuda index (MI)] were calculated using glucose and immunoreactive insulin (IRI) data from the 75 g OGTT, as previously described [12]. As indices of insulin secretion, the ratio of the area-under-the-insulin-curve to the area-under-the-glucose-curve during 0–120 min of the OGTT (AUC_ins/gluc_), which reflects glucose-induced insulin secretion in not only early phase but also in late phase [13] and the insulin secretion-sensitivity index-2 (ISSI-2) [14, 15], an oral disposition index that reflects the compensatory insulin secretion capacity against decreased insulin sensitivity (increased insulin resistance), were also calculated. To calculate ISSI-2, the AUC_ins/gluc_ was multiplied by the MI. The IGI, QUICKI, and MI, but not HOMA-β, have been shown to be significantly different among the NGT, IGM, and DM groups [10].

### Statistical analysis

Normally distributed continuous data are presented as mean ± SD and non-normally distributed continuous data are presented as median value, 25th percentile value and 75th percentile value. Difference among more than three groups was determined by analysis of variance (ANOVA) for normally distributed continuous data, by Kruskal–Wallis tests for non-normally distributed continuous data, and Scheffe’s test was performed as post hoc analysis. The relationship between the parametric data was determined using the Pearson’s correlation coefficients analysis. In regression analyses, normally distributed log_e_-transformed (ln−) values were performed for ucOC, HOMA-β, IGI, AUC_ins/gluc_, ISSI-2 and MI. In the simple correlation between ln-IGI and clinical factors including age, sex (female = 0, male = 1), BMI, and HbA1c in the DM group, the BMI coefficient (r) was highest (r of age, sex, BMI, and HbA1c were − 0.069, 0.022, 0.363, and 0.011, respectively). Therefore, in the multiple regression analysis of the DM group, in which ln-IGI was a dependent variable and ln-ucOC was an independent variable, BMI was selected as an additional independent variable. P values < 0.05 were considered statistically significant.

## Results

### Biochemical profiles of groups according to glucose tolerance

The ucOC was not significantly different among the three groups (Table 1). Indices of insulin secretion including AUC_ins/gluc_ and ISSI-2 differed among the NGT, IGM, and DM groups (Table 1).

**Table 1:** The serum ucOC levels and insulin secretion indices in the NGT, IGM and DM groups

	NGT (n = 25)	IGM (n = 25)	DM (n = 25)	P
ucOC (ng/mL)	4.3 (3.1, 5.2)	4.0 (3.3, 6.6)	4.0 (2.6, 5.8)	0.9411
Ln-ucOC	1.407 ± 0.516	1.383 ± 0.817	1.389 ± 0.606	0.9914
AUC_ins/gluc_ (pmol/mmol)	29.7 (20.4, 51.4)	32.4 (19.2, 46.0)	22.0 (13.3, 31.7)	0.0295
Ln- AUC_ins/gluc_	3.452 ± 0.593	3.465 ± 0.573	3.046 ± 0.545*^,^**	0.0164
ISSI-2	181.9 (164.8, 219.5)	119.8* (103.3, 158.3)	77.7*^,^** (59.4, 85.9)	< 0.0001
Ln-ISSI-2	5.266 ± 0.314	4.869 ± 0.351*	4.286 ± 0.334*^,^**	< 0.0001

Median values are shown. The 25th percentile and 75th percentile values are shown in parenthesis

*ucOC* undercarboxylated osteocalcin, *AUC*_*ins/gluc*_ the ratio of the area-under-the-insulin-curve to the area-under-the-glucose-curve, *ISS1-2* the component measures of the insulin secretion-sensitivity index-2

* P < 0.01 vs NGT; ** P < 0.01 vs IGM

### Simple regression analyses

**Fig. 1:**
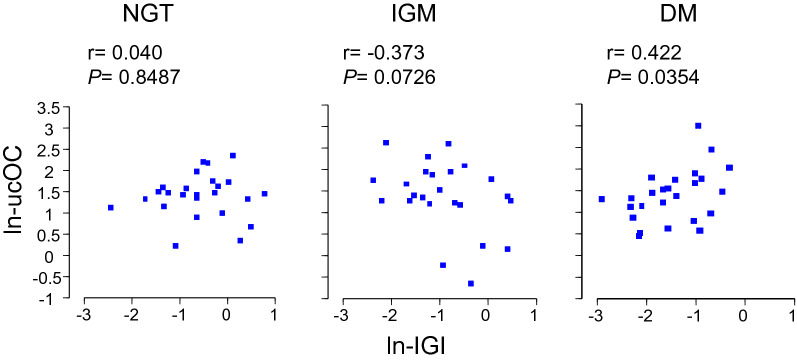
The relationships between ln-IGI and ln-ucOC in the NGT, IGM and DM groups (n = 25 in each group). *Ln-ucOC* Log_e_-transformed undercarboxylated osteocalcin (ng/mL), *Ln-IGI* Log_e_-transformed insulinogenic index [(μU/mL)/(mg/dL)]

Ln-ucOC did not significantly correlate with age, sex, BMI, waist circumference, fasting plasma glucose, plasma glucose 120 min after glucose loading, fasting plasma immunoreactive insulin, ln-HOMA-β, QUICKI, or ln-MI in any of the three groups (Table 2). Interestingly, ln-ucOC correlated with ln-IGI (r = 0.422, *P* = 0.0354), ln-AUC_ins/gluc_ (r = 0.397, *P* = 0.0491), ln-ISSI-2 (r = 0.536, *P* = 0.0057) and HbA1c (r = − 0.574, *P* = 0.0027) only in the DM group (Table 2, Fig. 1).

**Table 2:** The simple correlation between ln-ucOC and clinical factors in the NGT, IGM, and DM groups

	NGT (n = 25)		IGM (n = 25)		DM (n = 25)	
	r	*P*	r	*P*	r	*P*
Age	− 0.170	0.4174	0.280	0.1857	0.268	0.1959
Sex (F = 0, M = 1)	− 0.381	0.0606	− 0.141	0.5123	− 0.241	0.2456
BMI	0.395	0.0505	0.006	0.9783	− 0.366	0.0719
WC	0.295	0.1527	− 0.019	0.9315	− 0.136	0.5175
HbA1c (%)	− 0.048	0.8199	0.239	0.2608	− 0.574	0.0027
G_0_	0.275	0.1834	0.307	0.1444	− 0.392	0.0525
G_120_	0.302	0.1422	0.123	0.5662	− 0.222	0.2867
I_0_	0.138	0.5109	− 0.231	0.2773	− 0.249	0.2291
ln-HOMA-β	0.089	0.6722	− 0.332	0.1127	0.177	0.3961
ln-IGI	0.040	0.8487	− 0.373	0.0726	0.422	0.0354
ln-AUC_ins/gluc_	0.128	0.5406	− 0.281	0.1838	0.397	0.0491
ln-ISSI-2	− 0.233	0.2619	− 0.298	0.1577	0.536	0.0057
QUICKI	− 0.280	0.1756	0.082	0.7047	0.151	0.4718
ln-MI	− 0.264	0.2024	0.108	0.6155	− 0.069	0.7434

G_0_, and G_120_ indicate plasma glucose at 0 (fasting) and 120 min after glucose loading in 75gOGTT, respectively. I_0_ is plasma immunoreactive insulin (IRI) at 0 (fasting)

*BMI* body mass index, *WC* waist circumference, *QUICKI* Quantitative Insulin Sensitivity Check Index, *HOMA* homeostasis model assessment, *IGI* insulinogenic index, *MI* Matsuda index

### Multiple regression analysis in the DM group

Ln-IGI was independently predicted by BMI (β = 0.598, P = 0.0014) and ln-ucOC (β = 0.641, P = 0.0007) (R^2^ = 0.488, |R|= 0.699, *P* = 0.0006).

## Discussion

In the previous cross-sectional human studies, the 75gOGTT was not performed and insulin secretion was evaluated checking HOMA-β levels [7–9], and not by IGI. In a Japanese population, AUC for receiver operating characteristic (ROC) to predict the incidence of diabetes of IGI (0.825) is far larger than that of HOMA-β (0.604) [16]. Before the incidence of diabetes in women with gestational diabetes mellitus, IGI, but not HOMA-β, of individuals who had developed to diabetes is significantly lower than that of individuals who had not developed to diabetes [17]. Moreover, the utility of HOMA-β to predict diabetes development is questioned in nondiabetic Koreans [18]. These results indicate that IGI is a more reliable index of insulin secretion than HOMA-β in pathophysiological aspects. In addition, there are no previous reports showing the association between ucOC and glucose metabolism among groups classified into NGT, IGM, and DM groups with matched clinical backgrounds.

In this study, basal insulin secretion was not correlated with ucOC in diabetes as shown previously [19, 20]. However, we showed for the first time that serum ucOC is positively correlated with IGI, which reflects insulin secretion in early phase, in individuals with diabetes. Previous studies indicated that BMI was positively correlated with insulin secretion in individuals with diabetes, which might reflect compensatory augmentation of insulin secretion against insulin resistance [21, 22]. The present study also shows that ucOC correlates with IGI independent of BMI, thereby indicating it to be a novel factor to correlate with insulin secretion in individuals with diabetes. In addition, ucOC correlated with AUC_ins/gluc_ and ISSI-2, and this finding reflects the association between ucOC and insulin secretion in the early and late phases and accounts for the compensatory capacity against insulin resistance.

Interestingly, our results showed that ucOC was negatively correlated with HbA1c in the DM group. Since IGI did not correlate with HbA1c in the DM group in the present study, the correlation between ucOC and HbA1c via insulin secretion seems unlikely. In a previous study, chronic exposure to high concentrations of glucose showed a decrease in OCN gene transcription in osteoblast-like cell lines [23]. It can therefore be postulated that the suppressive effect on ucOC from osteoblasts, due to chronic exposure to high glucose, may play a role in impaired glucose-induced insulin secretion in diabetes.

Although basic studies demonstrated that ucOC affects insulin sensitivity, it did not correlate with indices of insulin sensitivity in any of the three groups in the present study (Table 2). In many literatures, total OCN including carboxylated OCN is weakly associated with insulin resistance [7–9, 24] for unknown reasons. The present results are compatible with separately shown results in which ucOC does not associate with insulin resistance in individuals with diabetes [20] and without diabetes [24].

### Limitations

The present study has limitations. First, since it was a cross-sectional study, a causal association could not be evaluated. It is unclear why the correlation between ucOC and insulin secretion was found only in individuals with diabetes. Second, since data were collected at a single institute in Japan, this study may have suffered a selection bias and therefore may not represent the broader population. Third, since the sample number is not large enough, weak correlations might be regarded as not significant and the influence of sex, which may affect ucOC levels, on the association between ucOC and insulin secretion was not analyzed in this study. Fourth, we did not measure plasma vitamin K levels. Prominent vitamin K deficiency is rare in the Japanese population except for patients with nutritional disorders and the individuals who were included in the present study did not have nutritional disorders and did not take warfarin. Since plasma vitamin K weakly and negatively correlates with ucOC in healthy Japanese women [25], vitamin K may also affect ucOC in IGM and DM. The influence of vitamin K on the association between ucOC and insulin secretion remains unknown.

## Conclusion

In conclusion, in our study, ucOC positively correlated with insulin secretion independently of BMI in Japanese individuals with diabetes. This suggests that in humans, ucOC may play more important roles in insulin secretion than in insulin sensitivity in individuals with diabetes.

## Data Availability

The ethics committee imposed restrictions to data access and sharing. Individuals who wish to access our data must obtain further permission from the committee, which can be achieved by contacting the corresponding author.

## Abbreviations

OGTT: Oral glucose tolerance test
IRI: Immunoreactive insulin
IGI: Insulinogenic index
HOMA: Homeostasis model assessment
QUICKI: Quantitative insulin sensitivity check index
MI: Matsuda index
AUC_ins__/__gluc_: The ratio of the area-under-the-insulin-curve to the area-under-the-glucose-curve during 0–120 min of the OGTT
ISSI-2: Insulin secretion-sensitivity index-2
ucOC: Undercarboxylated osteocalcin
ECLIA: Electro-chemiluminescence immunoassay
HbA1c: Glycated hemoglobin
NGT: Normal glucose tolerance
IGM: Impaired glucose metabolism
DM: Diabetes mellitus
G: Glucose
BMI: Body mass index
